# Supporting care home residents in the last year of life through ‘Needs Rounds’: Development of a pre-implementation programme theory through a rapid collaborative online approach

**DOI:** 10.3389/frhs.2022.1019602

**Published:** 2023-01-09

**Authors:** Aisha Macgregor, Brendan McCormack, Karen Spilsbury, Jo Hockley, Alasdair Rutherford, Margaret Ogden, Irene Soulsby, Maisie McKenzie, Barbara Hanratty, Liz Forbat

**Affiliations:** ^1^ Faculty of Social Sciences, University of Stirling, Stirling, Scotland; ^2^ Susan Wakil School of Nursing and Midwifery, The University of Sydney, Sydney, NSW, Australia; ^3^ School of Healthcare, University of Leeds, Leeds, England; ^4^ College of Medicine and Veterinary Science, University of Edinburgh, Scotland; ^5^ PPI lay Member; ^6^ Faculty of Medical Sciences, University of Newcastle, England

**Keywords:** care homes, co-production, engagement, end of life, theory development, palliative care, iPARIHS, hospice

## Abstract

**Background:**

Realist evaluation aims to address the knowledge to practice gap by explaining how an intervention is expected to work, as well as what is likely to impact upon the success of its implementation, by developing programme theories that link contexts, mechanisms and outcomes. Co-production approaches to the development of programme theories offer substantial benefits in addressing power relations, including and valuing different types of knowledge, and promoting buy-in from stakeholders while navigating the complex social systems in which innovations are embedded. This paper describes the co-production of an initial programme theory of how an evidence based intervention developed in Australia - called ‘Palliative Care Needs Rounds’ – might work in England and Scotland to support care home residents approaching their end of life.

**Methods:**

Using realist evaluation and iPARIHS (integrated Promoting Action on Research Implementation in Health Services) we sought to determine how contexts and mechanisms of change might shape implementation outcomes. Pre-intervention online interviews (*n* = 28) were conducted (February-April 2021), followed by four co-design online workshops with 43 participants (April-June 2021). The online interviews and workshops included a range of stakeholders, including care home staff, specialist palliative care staff, paramedics, general practitioners, and relatives of people living in care homes.

**Results:**

This methodology paper reports developments in realist evaluation and co-production methodologies, and how they were used to develop context, mechanisms, outcomes (CMOs) configurations, and chains of inference. The initial (pre-intervention) programme theory is used to illustrate this process. Two developments to iPARIHS are described. First, involving stakeholders in the collaborative co-design workshops created opportunities to commence facilitation. Second, we describe developing iPARIHS’ *innovation* component, to include novel stakeholder interpretations, perceptions and anticipated use of the intervention as they participated in workshop discussions.

**Conclusions:**

This rapid and robust co-production methodology draws on interactive collaborative research practices (interviews, workshop discussions of data, illustrative vignettes and visual methods). These innovative and engaging methods can be packaged for online processes to develop, describe and interrogate the CMOs in order to co-produce a programme theory. These approaches also commence facilitation and innovation, and can be adopted in other implementation science and realist studies.

## Introduction

Care homes are an increasingly common place for people to spend their last months of life due to dependency, frailty and illness (1, 2). Ensuring equitable access to high quality palliative and end of life care is important to ensure residents can live well until they die, with biopsychosocial needs anticipated and met, so that they can experience a good death. There is therefore a need for evidence-based approaches to support older people at the end of their life, and to reduce avoidable (and often detrimental) admissions to acute care settings.

One model of providing specialist palliative care expertise, which has been tested in Australia, is Palliative Care Needs Rounds (hereafter referred to as Needs Rounds). Needs Rounds combine (i) monthly triage meetings to discuss residents at risk of dying without a plan in place; these meetings follow a published checklist (3) and can trigger the second and third components of the approach, namely: (ii) multidisciplinary and/or family meetings involving advance and anticipatory care planning, and (iii) direct clinical work with residents, including medication reviews and symptom management. In Australia, Needs Rounds led to substantial cost savings (4, 5), enabled a greater number of residents to die in their preferred place (6), and improved staff confidence to look after people approaching end of life (7).

This study uses implementation science methodology and co-production methods to develop a mid-range theory for Needs Rounds to explain what works, for whom, in which circumstances, and support transferability of the successful model developed in Australia to an approach suitable for care homes in England and Scotland. Four case study sites are located in England and two in Scotland, with each site supporting up to six care homes (*n* = 29). This paper reports how realist evaluation and co-production were used to generate an initial programme theory prior to the intervention.

### Co-production and critical realism

There is a growing international focus on the impact of research on policy and practice (8) which has been reinforced in national quality frameworks (e.g., 9). It is no longer acceptable to consider academic publication as the endpoint of research activity, and funders often require that the pathway to impact is described and costed as an ongoing commitment to the translation of research findings into practice. This shift in research policy has also been driven by people living with a variety of long-term conditions who refuse to ‘be done to’ without active participation in decision-making about how services are provided. This ensures that lived-experience is central to decision-making (10) and has led to a proliferation of more collaborative approaches (11, 12). As a reflection of this position, research methodologies that privilege engagement with the communities or populations studied are increasingly used (11, 12). The language of co-production and co-design have become more commonplace and adopted as an ethical approach to research (13, 14). Co-production does not specify any particular method of research, but instead focuses on shifting the balance of power by ensuring that the end-users of research outputs and outcomes are active participants in each stage of the research design, operationalisation, dissemination, and implementation:

“Co-production refers to a way of working where service providers and users, work together to reach a collective outcome. The approach is value-driven and built on the principle that those who are affected by a service are best placed to help design it.”(15)

Although being committed to the co-production of research is a laudable aim, engaging in a co-design process at all stages of a research design is complex. Levels of participation range from being informants or recipients at one end of the spectrum, through to working as co-researchers where knowledge is co-produced (16, 17). The term co-production was originally coined in relation to the development of public services to mean “the process through which inputs used to produce a […] service are contributed by individuals who are not ‘in’ the same organization”(18 p1073). Although there is no consensus over a definitive definition of co-production (19), a number of key tenets are visible in the literature. Instead of being passive recipients, co-production involves individuals being both active producers and consumers of knowledge, and thereby having more control over operational decisions in research and/or practice (20). Co-production seeks to address power differentials, fostering greater equality and mutuality, where lived experience is given voice (21). This experience can help to surface new knowledge, and alongside improving relationships, the insights from people with lived experience can improve the chances of research being used in practice (17). Co-production has potential to be transformative (22), disrupting traditional research practices so that services meet the needs of users (21). Best practice suggests that participation should occur at all stages of the research process and take the form of a research partnership (20, 23).

Divergent influences have shaped how co-production is enacted. Co-production has been impacted by both a consumerist agenda, predominantly top-down, consultation based, and focused on service improvement, as well as more democratic approaches that seek to transform relationships to create more equal, reciprocal ways of working so that individuals have more choice and control (11, 24, 25). One of the key challenges is how power is distributed, and how different forms of knowledge are understood as equally valid. Conflict can arise if collaborators have different priorities, values, beliefs, and agendas (17, 20). Rather than being an empowering or transformative process, involvement and engagement can result in feelings of powerlessness (17).

Participatory methods are either explicitly or implicitly grounded in critical social theory where the emphasis is on ensuring that the views and concerns of marginalised people are represented (26). Critical social theorists hold the ontological position that no part of a social situation can be properly understood unless its historical and structural contexts are explicated. This ontology connects directly with the emphasis on co-production and co-design in research which include considerations of domination, power inequities, political contexts and oppression.

Critical realism provides the philosophical basis for critical social theory and the realist methodology adopted in the study reported in this paper. Critical realists argue that the real world operates as a complex multi-dimensional open system (27) and that generative mechanisms – powers, structures and relations that elucidate actions beneath what is observable (28) may remain suppressed until they are triggered within a particular context. Thus, we can never predict the outcome of a particular intervention as generative mechanisms produce ‘tendencies’ for action and the job of the researcher is to map those tendencies to determine the potential action of an intervention in a social context. It is from this epistemological perspective that realistic evaluation was developed (28), ensuring that the complex nature of social context is explicated in evaluation designs and moving away from a simple cause and effect mode of inquiry.

Koerner and colleagues demonstrate how successfully implementing Needs Rounds in Australia involved interactions between mechanisms operating in their inner and outer context to improve end of life care in aged care facilities (29). Contextual factors included readiness for change, leadership, staff knowledge and skills, and wider organisational policies. Mechanisms triggering change were care home and palliative care staff facilitation, identifying and triaging residents, strategising knowledge transfer, and changing clinical approaches to care through case conferences and anticipatory prescribing, planning documentation, and communication. This resulted in better preparedness and symptom management, reduced unnecessary hospitalisations, improved staff skills and confidence, and enabled better death and dying for residents. Reflections on similarities and differences between the Australian and UK context will be discussed later in this paper (29).

While realist designs emphasise engagement with all relevant stakeholders to ensure the inclusion of all potential mechanisms, the methods to achieve this are less clear. Indeed, it could be argued that the focus of stakeholder engagement operates more at the level of ‘involvement’ rather than at the more inclusive practice of engagement (30) or co-production.

The study described in this paper was explicitly designed to co-produce a programme theory, and involved throughout a range of people with relevant lived experience with opportunities to participate in diverse activities.

## Methods

This study's realist approach combines *the integrated Promoting Action on Research Implementation in Health Services* (iPARIHS) framework (31) and co-production methods to develop an initial theory on what works for whom to adapt Needs Rounds for care homes in England and Scotland.

### Study aims

• To collaborate with key stakeholders to co-design a model of Needs Rounds that is responsive to different contextual factors that are likely to impact successful implementation in England and Scotland.
• To devise initial programme theories about how change is expected to happen to produce the desired outcomes - about what might work (programme components and its evidence base) for whom it might work (key stakeholders) and in what circumstances (contextual factors).

#### Design

Sequential multiple methods qualitative study (32), with three stages. Stage 1 employed qualitative interviews, Stage 2 involved workshops, and Stage 3 used post workshop theory development sessions.

### Setting

Scotland and England, focused on six case study sites (where a case is a specialist palliative care team, and 4–6 care homes local to those palliative care teams).

### Sample

Twenty-eight people participated in interviews (individual or dyadic) from six case study sites prior to Needs Rounds commencing. Interviewees were key stakeholders: relatives, clinicians/managers in care homes, clinicians in specialist palliative care and related acute/primary care, ambulance staff, and pharmacists. Workshop participants (*n* = 43 unique participants attending between 1 and 4 workshops, from 23 organisations) were drawn from the same six case study sites, and included nurses, care assistants and managers from care homes, clinicians and managers from specialist palliative care teams, and patient/public involvement and engagement (PPIE) representatives. Although care homes were asked to invite residents to take part, we received no response from residents. This is likely due to a combination of gatekeeping, and the complexity of residents' compromised cognitive status/health needs, meaning many would be unable to take part (33, 34).

### Data collection

In stage 1, interview data were collected between February and April 2021 *via* online video platforms (MS Teams/Zoom) or phone. Individual and dyadic interviews were conducted and focused on contextual factors that are likely to impact on Needs Rounds, such as services' geography, policy, structure, funding and practice elements. The questions were informed by Estabrook's Alberta Context tool (35) to collect data on the care home type and funding model, areas of care provided, geography, education and service development, relationships (between care homes, specialist palliative care, acute care, and primary care), organisational policies, leadership and culture, and staff skills mix and confidence levels.

In stage 2, four co-design workshops were run on Zoom in April and June 2021. Illustrative questions are contained in the Supplementary File. This was selected as care homes were familiar with this platform, and to enable participation of a geographically diverse population during COVID-19. The contextual findings from the stage 1 interviews were presented to participants during the first and second workshops and discussion focused on whether this reflected their experiences, any identified gaps, and the anticipated mechanisms of change required for Needs Rounds to be successfully implemented. Data from both the stage 1 interviews and the following workshops were then used to create contexts, mechanisms, and outcomes configurations for five sub-theories. Fictionalized vignettes were then developed by the research team for each sub-theory and were presented during the third workshop. Discussions from the third workshop focused on overarching themes and connectors between the sub-theories. Discussions were captured by the investigator team (including academics and PPIE representatives) using traditional pen/paper as well as online whiteboard/note taking platforms (padlet). All discussions were audio recorded.

In stage 3, we built on the data from the interviews and three prior workshops in post workshop sessions to develop the programme theories. These were attended by the research team, which included PPIE members and academics with lived experience of supporting relatives in care homes and/or at the end of life. Members discussed the chains of inference connecting the sub-theories and the over-arching programme theory, and reflected upon how their academic, clinical, and lived experience impacted their interpretations.

### Analysis

Inductive thematic analysis was applied to data from stage 1 and 2 between February and June 2021 (36), using Nvivo to support coding and organisation of the data (see Table 1). Analysis focused on identifying the contexts (in which Needs Rounds will run), mechanisms of change, and outcomes (referred to hereafter as CMO). Contexts were analysed deductively as either inner context (individual or organisational level), or outer (such as the wider policy/cultural context). Mechanisms of change were organised into categories to include facilitators (people), facilitation (process), resources and reasoning. Innovation focused on understandings of Needs Rounds, perceptions of value, and degree of fit.

**Table 1:** Coding and themes.

iPARIHS component	Theme	Sub-theme
Outer (macro)	Care home sector & workforce	Absence, turnover & use of agency staff
		Diverse nature of the care home sector
		Pay, conditions, & opportunities for development
		Staff levels & ratios
	Commissioning	Commissioning process
		Funding levels
		Priorities & services commissioned
		Structure of health and social care system
	COVID	Access
		Capacity & demand on services
		Format of delivery
		Impact on care home sector
		Impact on relationships
		Impact on space
		Outbreaks
		Risk
	Policies & legislation	Anticipatory medications & medicines management
		COVID
		Enhanced Health in Care Homes Framework
		Verification of death
		Regulation
Inner (meso)	Attitudes, culture & leadership	Attitudes of leaders
		Attitudes towards care home staff/work
		Culture & leadership
	Policies & processes	Care home policies
		Hospice policies
	Relationships	Communication (accessibility, accuracy & clarity, format, information sharing)
		Engagement (attendance, format, reach)
		Multi-disciplinary working (fractured, fragmented/poor relationships; ownership; professional knowledge & expertise; proactive/partnership working; collaboration & empowerment; responsiveness & time)
		Supporting families
Inner (micro)	Care giving/doing care	Acute care (discharge planning, reasons for hospitalisations)
		Ambulance care (confidence, capability & training; emergency response; non-emergency, time; transporting to hospital)
		Care homes (resources needed for implementation; confidence and competence motivation to change/buy in; past experience of innovation & change; workforce issues)
		Hospice (services; space; staff mix)
		Primary Care (district nurses; GP service provision; pharmacy)
	Care Quality	Clinical & personal care
		Compassion, dignity & respect
		Personalised
		Relationships & knowledge
		Safety & risk
	Demographics	Ethnicity
		Geography
		Malignant/non-malignant
		Socio-economic levels
Innovation	Understanding	CH staff well informed of Needs Rounds
		Understanding amongst wider stakeholders/organisational networks
	Degree of fit	Strategic priorities in relation to palliative and end of life care
		Ethos of training, education, and quality improvement
Recipients	Perception of value	Motivation to change/buy in (amongst CH staff (and directors/senior management in chains), SPC (clinicians and senior management/trustees), primary care, ambulance staff, acute care)
Mechanisms of Change	Facilitation	Developing trusting, reciprocal relationships (safe space and mutual respect & recognition)
		Addressing power hierarchies
		Negotiating and influencing
		Organising care home staff for attendance (rotating to ensure the rights kills mix and knowledge of the resident)
		Liaising with external stakeholders (eg GP where existing NRs)
		Buddying & mentoring
	Facilitators	Micro - Care home leads – registered nurses, heads of unit, senior nurses, clinical leads, care team member with best knowledge of resident
		Meso - Care coordinators, clinical governance committees, trustees
	Resources	Time
		Training & education (structure within CH & case based education, integration with existing education mechanisms)
		Care planning and clinical actions (advance care planning, medication reviews, anticipatory medications & de-prescribing), symptom control & pain management)
		Standardised yet contextualised information sharing (template to be used within existing systems, utilising existing documentation used in area (ag ReSPECT), tailored, clear communication
		materials of the benefits of NRs to secure buy in; communication with policy makers/commissioners)
		Payment (access to holistic services)
		Space (for NRs to prevent disruption)
		Alignment with existing proactive work (eg MDT meetings, weekly rounds, GSF meetings to prevent duplication & strengthen relationships)
	Reasoning	Better understanding of how to communicate with professionals and relatives
		Care home ownership of NRs
		Choice of staff attending NRs
		Competence to recognise deterioration
		Increased staff confidence
		Visibility (collective view amongst stakeholders, formal & informal meetings)
Outcomes	Staff confidence & competence	Improved staff confidence in advance care planning (goals of care, ceilings of treatment and place of care, anticipatory medications)
		Improved communication amongst care home staff and professionals, residents, and relatives
		Improved recognition of deterioration and dying
		Care homes perceived as experts in palliative and end of life care
	Improved inspection ratings	Better documentation
		Evidence of quality improvement
	Better support to families	Families involved in care planning
		Better relationships with families; less conflict
		Families feel confident in staff ability to care for their loved ones
	Improved inter-disciplinary working	Reduced inappropriate contact with GPs
		Improved discharge planning
		Improved knowledge and understanding of professional roles & respecting boundaries
		Strengthened relationships between care homes and hospices
	Better quality palliative and end of life care for residents	Proactive advance care planning and documentation – goals of care, ceilings of treatment, dying in preferred place
		Proactive anticipatory medications & deprescribing reviews
		Better symptom control and pain management
		A good death, reflecting resident wishes.

Coding and analysis were commenced by an experienced qualitative health researcher. Co-investigators (with a range of qualitative, quantitative, academic and lived experience) discussed data and interpretation to refine analytic codes and to develop initial sub-theories and a proposed over-arching programme theory. The involvement of other stakeholders in the analysis and theory development is described in the results section.

### Ethical approval

The study was approved by [Frenchay Research Ethics Committee, ref: 287447]. Formal respondent checking of transcripts was not used, since the workshop provided opportunity to clarify, check accuracy and validate ideas/opinions with participants.

### Positionality

Three of the co-investigators are PPIE members, and five members of the research team are academics with lived experience of supporting relatives in care homes, and/or palliative care. Thus, the co-development with participants recruited to the research because they are employed by care homes or specialist palliative care, was enriched by many of the co-investigators straddling identities, holding both personal and professional connections to the study aims.

This positionality disrupts erroneous constructions of dichotomous insider and outsider identities, with the research team occupying positions of both academic and lived-experience (37, 38). Including both standpoints, enables both rigorous analysis and the scrutiny of research processes, data and outcomes (39).

Manzano promotes moving beyond an insider or outsider identity in realist evaluation interviews, instead adopting a theory driven approach based on the ‘learner-teacher cycle’ (40). Rather than being ostensibly neutral, researchers and participants are encouraged to engage in a reciprocal relationship where all parties participate in learning in order to develop and refine the programme theory being tested. This study sought to build on this collaborative approach not only in relation to *testing* the programme theory, but also with regards to its *development*.

## Results

**Figure 1:**
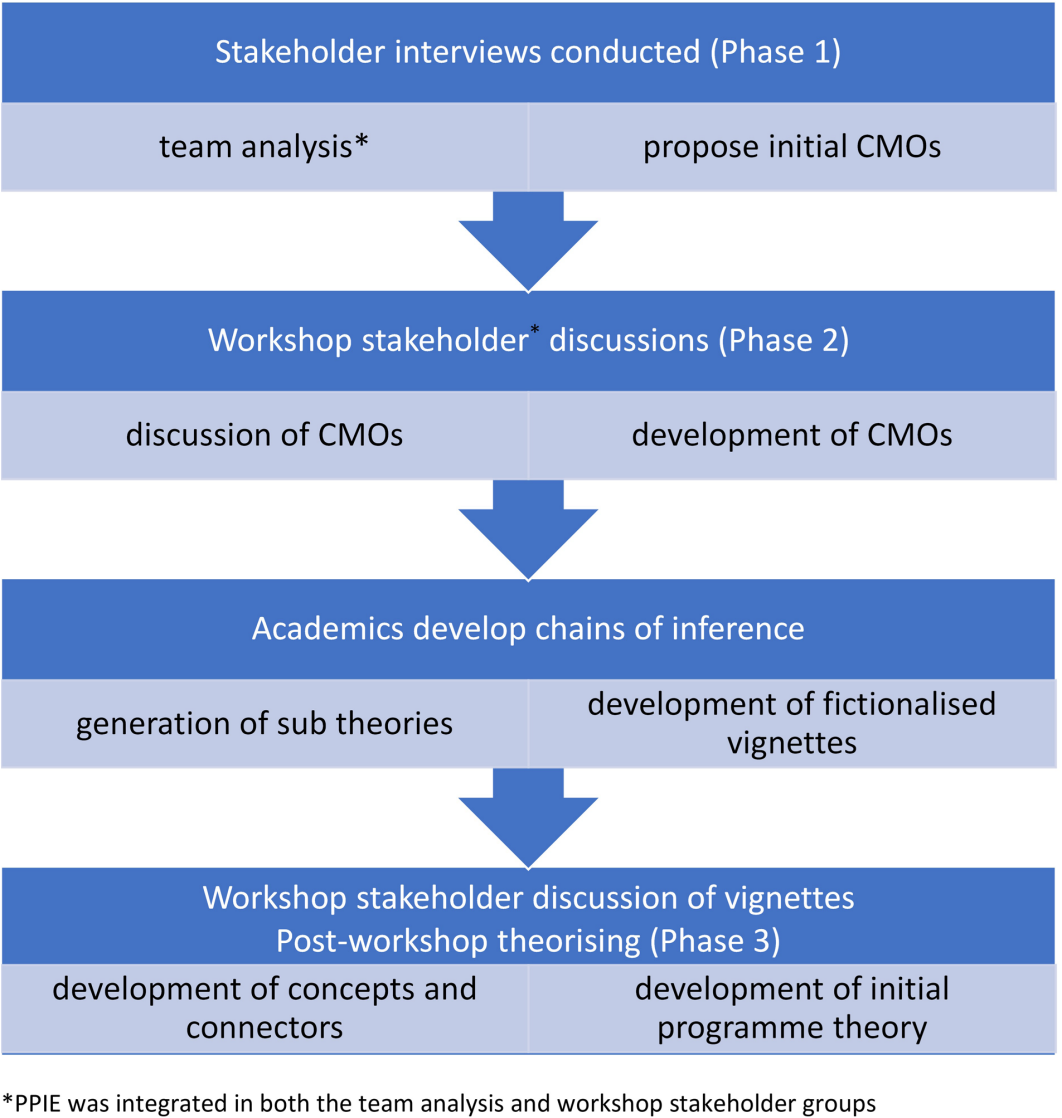
Iterative co-production methodology.

We developed an initial programme theory through an iterative co-produced methodology as illustrated in Figure 1. We show how the movement between three modes of participation in pre-intervention theory development (i semi-structured interviews, ii stakeholder engagement workshops, iii post-workshop theory development sessions) enabled the generation of an evidence base that was collaboratively and systematically refined through layers of facilitated engagement. Each of these modes of participation is also a mechanism for facilitation and enabled the innovation to be adapted to reflect novel stakeholder perceptions during the design stage. These engagement processes are described using one sub-theory, in the initial programme theory, as an exemplar.

### Robust and innovative codesign techniques with stakeholders

The innovation construct within iPARIHS recognises that evidence is often adapted in line with recipients' underlying knowledge sources, including their contexts, motivations and perceptions regarding the innovation (31). The overarching co-production approach was used as an innovation to elucidate a range of stakeholder views, understand how Needs Rounds fit with existing care home organisational processes, and explicate how these intertwine with external systems. As will be described, the knowledge generated in stages 1–3 enabled the UK model to be adapted to reflect the wider context.

Interviews generated content that was used in the collaborative co-design workshops. Interviews with stakeholders also marked the start of engagement with people who would be able to act as intervention facilitators during the subsequent (implementation) phase of the study. Workshops proceeded with a mixture of large group discussion, and small working-group breakout sessions with the stakeholders (described in the methods section). While co-production commenced pre-funding (with co-investigators being people with lived experience of care homes), the workshops formally engaged with people working in the health and social care sector supporting people living in care homes. Table 2 summarises the aims and objectives of each of the four stakeholder co-production workshops. The content and process of Workshop 4 is described in another paper, since the final workshop focused on provision of training rather than development of an initial programme theory.

**Table 2:** Co-design and CMO development workshop aims and objectives.

Workshop number and title	Aims	Objectives
1: The big picture	Develop a shared understanding of what Needs Rounds are, their evidence base and the differences with the Australian context.	Small group discussion of initial outer level contextual themes from stage 1 interviews and how these impact upon different stakeholder groups.
	Use the discussion from today to contribute to generating and refine a theory of change.	Large group feedback and reflections on the initial analysis to promote consensus and questioning.
2: It's all about you, baby	Focus on the context factors and mechanisms of change for stakeholders, to generate a first version of a CMO model^1^.	Present interview data on the inner context data from stage 1 interviews and how these impact upon different stakeholder groups and systems.
	Use the discussion to continue to generate and refine a theory of change.	Discuss sites’ motivation and ability to change, the fit of Needs Rounds with organisational priorities, and how Needs Rounds should be operationalised to fit the local context.
		Determine what implementation of Needs Rounds will look like at inner/outer levels, and how we anticipate inner/outer contextual factors influencing the mechanisms of change.
3: The UK's next top model	Articulate theory of change in relation to i-PAHRIS facilitation (facilitators as people and facilitation as process), innovation (Needs Rounds components and theory around diffusion) (30), recipients (key stakeholders) and context (inner and outer)	Present the relationships between different elements of the CMO using fictionalised narrative vignettes.
	Link theory of change to core theories which underpin implementation science/IPARHIS (experiential learning, situated learning, evidence-based practice and innovation). (30)	In small group discussions, engage participants in dialogue to determine how the theory fits with prior discussions and their local context, and identify any gaps.
4: Ready, Set, Go!	Ensure all sites are prepared to commence implementation, with specialist palliative care clinicians trained in their role, and care home staff prepped and ready to start.	1. Provide training in Needs Rounds and discuss practicalities for commencing implementation. 2. To simulate a Needs Round, based on recent/current examples from care home participants and guide learning on: i. the triggers for bringing someone to a Needs Round ii. managing the pace and engagement required to run Needs Rounds to time iii. effective planning for Needs Rounds, e.g. room/time/med charts 3. iv. formalising methods of embedding change, encouraging learning and development.

**Figure 2:**
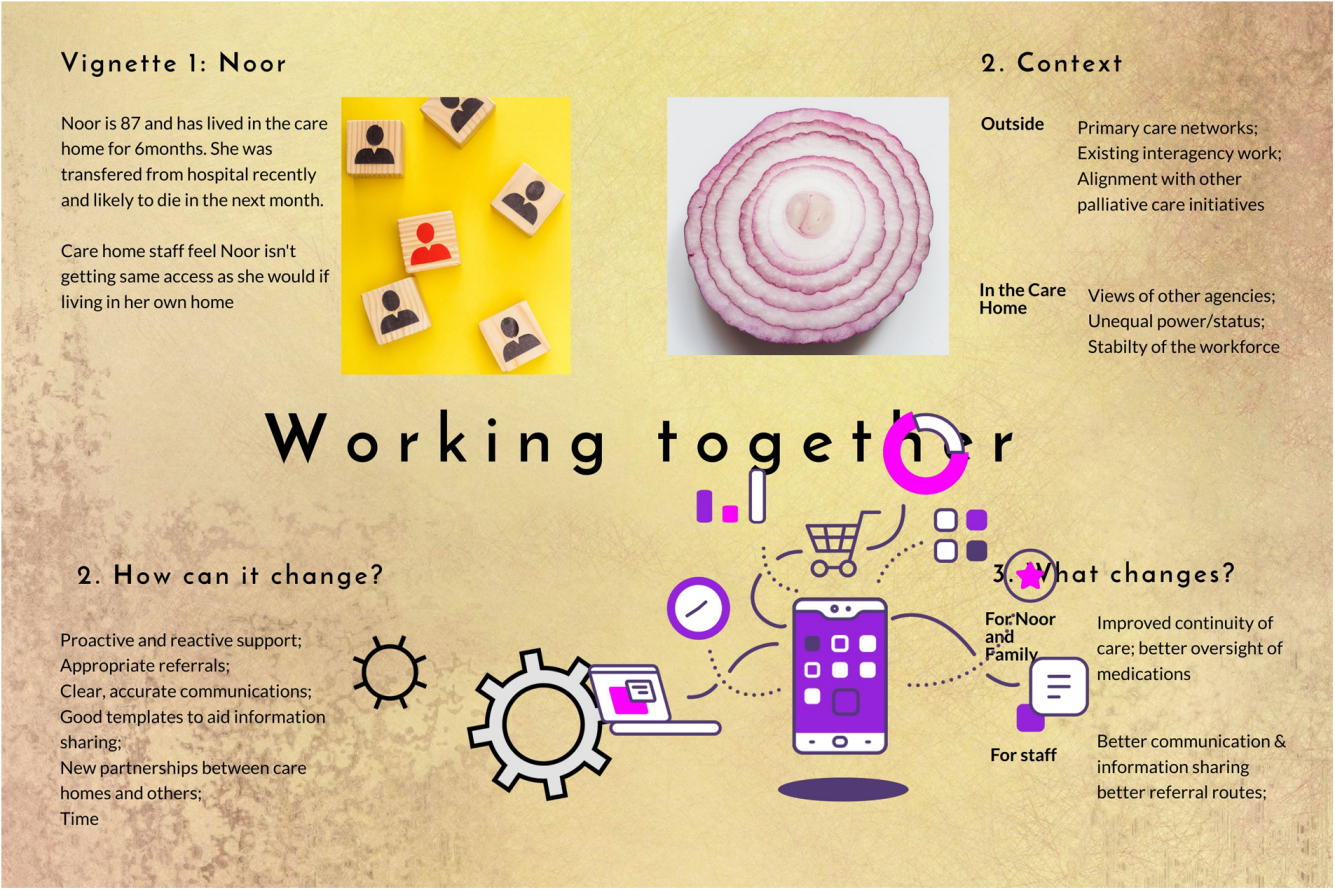
Fictionalised vignette of ‘working together’.

Briefings summarising interview findings from stage 1 were presented to all workshop participants during the first and second workshops using a traditional PowerPoint format. These focused on inner and outer contextual factors that had been identified in the stage 1 interviews. From the interview data and feedback from the initial workshops, chains of inference were then developed by the research team, describing the links between contexts, mechanisms and outcomes (summarised in Table 3). These chains of inference were then further developed and represented in one of the fictionalised vignettes (Figure 2), and then refined as a sub-theory called ‘working together’. The vignette focusses on a care home resident with complex needs who did not have access to specialist palliative care.

**Table 3:** Interagency working theory chains of inference.

Context	Mechanisms	Outcomes	Illustrative data
To build trusting relationships based on an equal partnership, where the knowledge and expertise of partners are valued and respected	Partnership working, based on reciprocity and collaboration (process), between specialist palliative care clinicians & CHs (people).	This may strengthen relationships and improve mutual understanding between hospices and care homes, leading to better engagement by care home staff in Needs Rounds and improved uptake of wider specialist palliative care beyond Needs Rounds.	“I think what works well is when we have good relationships with the teams that we’re working with, and that we’re listening to them, and we’re totally on board with what they’re wanting […] when we’re all learning together, which we are, and it's that reciprocal relationship, I think that's when it works well […] we don’t want to be going in and telling people what they need to know. It's about relationships and partnerships.” SPC1002
To empower care home workers who feel undervalued and unappreciated.	Care homes feel a sense of ownership over Needs Rounds, with support through shared facilitation and expertise (process).	Care home staff feel engaged and motivated to deliver Needs Rounds and drive forward change	“Care home staff can feel very got at sometimes, that people are very punitive, you did that wrong, you should’ve done that” SPC1004
For care home staff, the specialist palliative care clinician(s), relatives/residents (where have capacity), GPs, and other relevant professionals (eg. psychiatrists, pharmacy, geriatrician) to address challenges created through high turnover/poor continuity of care home workers (outer), and negative attitudes towards care home staff.	Structured time through monthly Needs Rounds (resource) and follow up multi-disciplinary team meetings for advance care planning, based on a collaborative approach (process).	This may improve the quality of advance care plans, ensuring these reflect resident and relative preferences, and lead to better access to anticipatory medications, to prevent reactive decision-making and unnecessary hospitalisations. This may also improve joint working/the development of relationships through better communication, trust and sharing expertise, clarity around roles and responsibilities, preventing duplication, and professionals taking ownership for their clinical area of care.	“It is particularly challenging and complex work to be working within the care home sector. I think the type of work that care home staff do is not well acknowledged and appreciated within society. I think rates of pay are poor, I think opportunities for promotion and development can sometimes be limited, and for these reasons people will- are more likely to move on to other jobs” GP1018
For CH staff with low confidence in communicating with professionals, leading to blockages in communication.	Case-based education (process) on effective communication with professionals.	Improved communication, based on the provision of accurate, relevant, up to date information.	“A lot of it is about empowering them I think, and being able to give the right information, so that they can then persuade the person that actually, this is important, and I need to come” SPC1002
For CHs, acute care, primary care, hospices, and other relevant professionals and aligning with existing mechanisms for anticipatory care planning/reviews.	Advance/anticipatory care plans & care records (resource) documented, communicated, and stored using the preferred local information sharing system in a timely way (process).	Up to date and accurate understanding of resident's history and care wishes may enable care to be delivered in a way that reflects their preferences. This may also help to build trust and improve inter-disciplinary relationships.	“I would recommend that Needs Rounds are done as part of the enhanced health in care home's meetings…we’ve got this relationship we’re building with continuity of care, we’re looking at anticipatory prescribing. We’re red coding. We’re talking about planning. I'm meeting with the families. If the Needs Rounds then says setup on the Friday and said, we’ve talked to the family, I’ll be thinking, “What?” I would find that intrusive. However, some of that isn’t helpful. We have to bring that in. But I think you have to work with the continuity and so I would find it quite jarring if they didn’t work with us.” GP1015
Primary care professionals varied in their responsiveness to CHs, although hospices perceived as being very responsive and willing to help.	Multi-disciplinary team meetings following Needs Rounds to draw in a wider multidisciplinary team (process).	Enhanced medicine management of CH residents with dementia, and enhanced symptom management.	“GPs, depending on whether it's in hours or out of hours it can be a little bit hit and miss. But again, we don’t know exactly when they’re going to come” A1009
For regulators focused on outcomes and the use of data in evidencing high quality care standards.	Evidence on the quality of death and dying and staff confidence and competence to support people at the end of life (resource).	Improved documentation of evidence-based practice for CHs, leading to improved regulator inspection assessments.	“It does seem to be that they’re [regulators] focusing a lot on data rather than inspection visits, so you know feeding in from what was just said, I think the data that's coming out with the end of life quality assessments will be really helpful for the providers, in providing evidence that's requested from the CQC” Workshop 1, breakout 1, SPC.

Abbreviations: A, ambulance service; CH, care home; GP, general practitioner; CQC, care quality commission; SPC, specialist palliative care.

The fictionalized vignettes were used during workshop three to facilitate workshop participants' engagement with discussion of the thematic analysis. These were an innovation to aid workshop participants to engage with the contexts, mechanisms and outcomes identified through analysis of the interviews and data from the first two workshops. The vignettes adopted a narrative story-telling approach to ensure that the complexity of language and theory in implementation science was not a barrier to participating in theory development. The vignette uses the metaphor of onion rings to examine the inner and outer layers of context (micro, meso and macro factors) which influence resident care. The moving cogs depict the mechanisms that need triggering to effect change. The final component of the vignette illustrates how the context and mechanisms can lead to desired outcomes (what changes) for the resident, family and staff. Further to these approaches, a small number of participants were nominated as ‘guardian angels’ whose role was to speak out if jargon or complexity was becoming a barrier to full engagement.

Small breakout sessions were used to a) understand if the vignettes reflected participant experiences and b) explore any gaps in knowledge about the contexts, or mechanism of how Needs Rounds might work in practice. Group discussion sessions focused on stakeholders identifying overarching themes, and participants identified and described connectors between these themes.

Workshop participants and PPIE members reflected that the use of fictionalized vignettes aided understanding. This was perceived as being more accessible than using implementation science language and traditional powerpoint. As one PPIE member noted:

“I don't come from a health or academic background, and I found the onion rings an excellent way of showing how the layers worked. For me, it was easier to visualise, it was easier to grasp the complexities of interaction. The vignettes, I felt, showed the complexities of caring and what a resident might look like”(PPIE member 1, reflective diary).

The use of fictionalized vignettes was therefore an innovation designed to broaden understanding of the complex methodological language, stage 1 evidence, and notions of contexts, mechanisms and outcomes amongst recipients. A worked example is provided of one of the five sub-theories generated from the interview data and stakeholder workshop discussions in Table 3. The CMO configurations are noted, alongside quotations taken from stakeholders engaged in the stage 1 interviews and stage 2 workshops. The role of facilitation is implicit within the mechanisms. Active engagement of stakeholders in the workshops meant that the role (facilitator) and activities (facilitation) were identified, described, and ascribed to individuals at this early pre-intervention stage.

During post workshop theory development sessions, the five sub theories were refined, including their chains of inference, and how they connect to produce one initial overarching programme theory. The importance of communicative spaces, for example, was explored, where dedicated space is created for people to feel safe and brave to reflect and contribute based on their knowledge of their residents and their needs. Interconnected issues were discussed, including power and hierarchy, whose voice is being heard, and how team and organizational cultures feed into this. One PPIE member, for example, said

‘[it's] about the culture of teams and organisations…. And it's this thing about balance of power and being brave enough and courageous enough… So for me that's the issue that's really key: dissemination, how we’re going to change culture, and listen to voices we are not always hearing’ (PPIE member 2, post workshop theory development session 1).

### Illustrative initial programme sub-theory: working together

One of the sub-theories focused on ‘working together’ and stated that: *an approach based on collaboration, empowerment and partnership (m) that challenges unequal power dynamics and builds trust (c) may lead to improved relationships and better joined up working (o)*. 

(where m = mechanism, c = context, o = outcome).

There was significant variation in the quality and nature of inter-agency working across and within the sites. Participants highlighted how suboptimal multi-disciplinary working is shaped by unequal relationships marked by issues of power and status. Attitudes towards care home workers can be negative, with care work being undervalued, which can impact upon care workers' confidence. Care home staff discussed how their intimate knowledge of residents can be disregarded, leading to challenges when seeking clinical support, such as transfer to hospital or changes to medication regimes. Some participants also experienced issues with the length of time it took to receive a response from the GP, particularly when dealing with out of hours services. Problems were described regarding doctors not taking ownership due to a lack of knowledge of residents and not wanting to conflict with the registered GPs recommended care and treatment plan.

Ambulance staff and GPs highlighted the challenging nature of working with care homes. Staff workforce issues including high turnover, sickness, and/or reliance on agency staff can restrict the development of knowledge of residents and their needs, and this can create difficulties during handovers. Skills deficiencies amongst some care home staff were also perceived to create friction between professionals, particularly when there was an expectation that they should be carrying out certain types of work. Issues were also raised around the quality of information being shared amongst professionals. A need for clear, accurate, and accessible information was emphasised, with blockages in information sharing – both systems and content – impacting upon resident care.

Some care homes had positive working relationships with external agencies, with continuity and time being critical factors in the development of these. Proactive, regular support from GPs was valued for enabling trust and knowledge of both staff and residents to be built, and in enabling the detection of subtle changes in symptoms and illness trajectories. Proactive support was sometimes facilitated through the Enhanced Health in Care Homes Framework (41) or a retainer paid directly to the surgery, which occurred in some sites where the majority of residents were privately funded. Hospices were highly valued by care homes and ambulance staff for their expertise, particularly in relation to residents with complex needs, and for being responsive, compassionate and willing to help, at any time of the day.

The sub-theory for ‘working together’ was thus shaped by participant discussions on the range of other palliative care supports for care home residents and inter-organisational networks to support Needs Rounds. The theory integrated Needs Rounds’ future sustainability, local and national information sharing systems for health and social care, and how multi-disciplinary working is organised across the sites. The theory attends to how anticipatory and advance care plans are shared, anticipatory medicines and de-prescribing processes, alongside how best to engage with local stakeholders to secure buy-in, including how GPs should be involved. During the first workshops, specialist palliative care staff were conceived as experts, whilst there was also a collective valuing of the different skills, knowledge, and expertise of care home staff. The need to work together collaboratively was reflected in feedback from the meso context breakout sessions, where participants summarised the discussion. They said:

‘The care home should own the needs round… they [specialist palliative care participants] fully supported that sitting alongside, working with the care home to meet the needs of the residents… there was a sense in which this should be co-facilitated, so yes led by the care home, but the teams sitting alongside each other to do the right thing at the right time for the resident… if the trigger was more around lack of food intake, or, it could actually someone on the housekeeping side who's around who knows a bit more about the resident and what their needs are at that time’ (academic with lived experience, workshop 1).

'They [care home staff] are the specialists actually because the nuances they know can make such a difference. They might not know the actual […] clinical terms, but the amount of detail that they know can make such a difference to somebody's life and the noticing that goes on that we don't actually capture.' (senior management (NHS), workshop 3).

Connectors between each of the vignettes (summarising each of the sub-theories) were developed in the workshops and were regrouped into larger categories (see Table 4) and these were refined to generate one over-arching initial programme theory about what works for whom in what circumstances with UK Needs Rounds.

**Table 4:** CMO connectors and themes.

Connectors	Themes generated from stage 1 and stage 2 data
**Trust**: creating a safe space for participants to be vulnerable about their needs/knowledge, and building trust through partnership working	Vulnerability in the context of a safe space can enable meaningful education; internal policy (trust or lack of trust to make autonomous decisions); partnerships and relationships; lack of trust in hierarchies; decision-making (care home staff trust own judgement to make decisions instead of referring out).
**Power**: understanding how power is distributed within relationships; Needs Rounds facilitation will flatten any unequal power dynamics and empowering care home staff to have voice	Empowerment, brave space, partnership and collaboration, reciprocity, sensitive leadership, hierarchies, autonomy, decision-making, and expertise, voice (different voices and how these are heard), regulation (care homes not viewed as experts - Needs Rounds provides evidence), greater regulation based on funding type (e.g. more for publicly funded compared to privately funded residents), and care home type (e.g. if part of a chain can be more restrictive).
**Communication**: trust and more equal power dynamics can foster good communication; developing mechanisms to improve communication will be a key part of facilitation (eg a resource mechanism being an information sharing template, education around best practice in high quality on advance/anticipatory care planning, documentation and sharing	Decision-making, trust between professionals (across and within agencies), residents, and relatives; resident wishes realised; families feel more informed, involved, and secure in their relative's care.
**Resident in their wider system**: a whole system approach to improve care quality; care homes are operating within complex systems and focusing solely on the individual risks obfuscating the context	Voice (of residents and relatives); regulation and legal frameworks on decision-making; high quality systemically situated proactive anticipatory/advance care planning; relationships with other health/social care providers.

From this sequence of interactive collaborative research practices (interviews, workshop discussions of data, illustrative vignettes, co-production of initial lines of inference, and development of CMO connectors and themes) we were able to generate a cohesive initial programme theory for testing. Innovations to the Australian approach to Needs Rounds were generated in response to discussions of contexts and mechanisms, which flowed through into the connectors and themes.

Contextual mapping identified through the interviews and workshops enabled adaptions to be made to the innovation. Whilst similarities exist between Australia and the UK in relation to workforce challenges, with both experiencing high staff turnover, and care home residents having complex multiple morbidities, four key differences are also evident. First, the size and scale of Needs Rounds operating across the two countries shaped the innovation. In the stepped wedge randomized control trial in Australia, Needs Rounds were delivered in 12 care homes in the Australian Capital Territory (5), compared to 29 care homes across England and Scotland. Implementing the intervention across two countries as opposed to one district, with different health care systems, legislation, policy, funding, and commissioning highlights the contextual complexity within which Needs Rounds are embedded.

Second, Needs Rounds were adapted in the UK to align with existing palliative care provision, including the Gold Standards Framework, and the Enhanced Health in Care Homes Framework, the latter of which only operates in English sites. Local contextual differences therefore had to be accounted for in the overarching programme theory. This was achieved by encouraging sites to align the Needs Rounds discussions with existing multi-disciplinary meetings, which did not happen in Australia.

Third, Australian care homes all employed registered nurses, whilst care homes with and without nursing care were included in the Scottish and English sites. As a result, it was anticipated that greater links would have to be made with primary care to ensure anticipatory prescribing and de-prescribing took place. Fourth, UK Needs Rounds took place during COVID-19, therefore flexibility in delivery mechanisms were built into the innovation, including both remote and in person options. COVID-19 also meant that staff were under prolonged pressure delivering care while managing outbreaks and lockdowns.

Our initial programme theory states:

Needs Rounds can provide care home and specialist palliative care staff opportunity to collaborate during a protected time to plan for residents' last months and weeks of life, by strengthening relationships and trust between care home staff, care home relatives, specialist palliative care staff and health services. Flattening unequal power dynamics, and strengthening communication mechanisms leads to high quality resident centred care which benefits the resident, relatives and staff.

### The value and structure of integrating PPIE

PPIE has been critiqued for being tokenistic, for failing to have a tangible impact on research processes, and because of the unequal power dynamics that shape involvement (42, 43). Facilitating high quality PPIE has been an integral part of the project from the outset to ensure that the development of Needs Rounds is informed by the views of people with lived experience. Indeed one of the outputs from this project will be an evaluation of PPIE experiences and processes to build learning for future studies and contribute to the evidence and understanding of PPIE (44).

PPIE members contribute by focusing the project on the perspective of family members and care home residents. They have shaped the project design through contributions to monthly investigator meetings, including influencing the research questions for the stage 1 interviews and the development plans for the stage 2 workshops. This included thinking about ways to ensure that the workshops were accessible, a pertinent issue given the technical terminology and conceptual nature of implementation science. PPIE input resulted in the proposed workshop plans being refocused to include vignettes to illustrate the CMO configurations (cogs, onion rings, and change) to promote understanding, and the use of ‘guardian angels’ to flag if the content was too technical. Following the workshops, PPIE members helped to further develop and refine initial programme theories. This included highlighting the importance of voice, power, and cultures of hierarchy, key connectors which were anticipated to impact upon how Needs Rounds would run.

Support was provided to PPIE members to ensure their full participation. For example, training on Needs Rounds and implementation science at the start of the project, practice-sessions on the online conferencing platform to familiarise themselves with the technology, and briefing sessions focused on the overarching workshop aims. Detailed briefing packs were also distributed in advance of each workshop to ensure PPIE members would be able to contribute their views, and also facilitate others to share their perspectives. PPIE members reported that this mix of preparatory activities meant they felt able to participate and contribute to discussions.

Investigators contributed their insights in post-workshop team discussions drawing on academic, clinical and lived experiences. Team members holding two or more of these expertise/experiences did so reflexively and by explicitly signposting the positionality they were adopting. For example, stating that their experience of their parent in a care home led them to think about the mechanisms they had observed within the sub-theories, alongside their prior use of implementation science prompting reflection on how facilitation might work.

Within this frame of acknowledging the multiple positions of investigators, we did not seek to determine specific outcomes from each speech act positioning self as expert-by-experience. Rather the multiple positions were harnessed as part of the emergent and dialogical process of guiding the study.

## Discussion

The development of initial programme theories pre-intervention is poorly described in the methodological literature (cf 45). This paper has provided a detailed description of the process of involving key stakeholders (by work role and/or lived experience) using online processes in the development of CMO configurations, chains of inference, and initial (pre-intervention) programme theories. The process and forms of engagement outlined contribute to the overall endeavour of ensuring that the programme theory generated was embedded within, and generated by, relevant communities. Co-production was an explicit *a priori* methodology which was resourced and planned for, throughout the study (46).

The paper also articulates two developments to iPARIHS. First, involving stakeholders in the collaborative co-design workshops created opportunities to commence facilitation. As iPARIHS and implementation studies have identified, facilitation is an integral element to the success of adopting new approaches in practice (31, 47, 48). Facilitation can be a role, process or structure (31, 49). The workshops provided a structure to engage key stakeholders in the study; providing a foundational approach to buy-in at the development stage, regarding the nuances of how Needs Rounds would work. Workshop participants also explored their understanding of their role as facilitators in delivering the intervention (i.e., care home and palliative care staff who would be running Needs Rounds). Consequently, the recruitment of stakeholders to the workshops acted as a mechanism to engage and prepare them as facilitators, months before the formal intervention commenced (31). Through engaging stakeholders and intervention facilitators, they were able to determine the parameters and pragmatics of second order change within their organisations.

Thus, the approach to workshop engagement generated both a process and structure for stakeholder facilitation to start prior to implementation. As noted in Table 3, people and processes, are core mechanisms for the delivery of the intervention.

The second development to iPARIHS is developing the model's *innovation* component, whereby evidence included prior studies' findings, but also novel stakeholder interpretations, perceptions and anticipated use of the intervention as they participated in workshop discussions. Creating space and a mechanism for stakeholder knowledge and PPIE from the study's commencement has introduced a new phase for conceptualising intervention recipients (31). The process of innovation will be completed during the implementation phase, and hence reported fully at the end of the study.

The co-production element invokes several well-documented challenges in PPIE, such as managing power differentials (50). While valuing all parties' contributions, the research team hold final power in proposing the final programme theory. Ensuring that the research team include people with lived experience (as well as straddling academic and lived-experience) presents an approach to ensuring that power is distributed and reflexive.

Realist epistemology has an easy fit with the ideology of co-production *and* the idea of open systems (51). To understand and work within the complexity of open systems, it is essential to include views of people within these systems, and the context and varying mechanisms within which implementation occurs. In doing this, we believe that our approach adopted a systematic way of dealing with what Bhaskar (51) refers to at the ‘epistemic fallacy’ – shifting our gaze to what we ‘know’ to be real, or exists, to that of working to ‘understand’ the nature of reality. This shift underpins the research policy drive underpinning co-production in research designs and prevents the reaching of conclusions that may not represent the actual nature of reality of end-of-life care in care homes. As critical realists argue, reality is comprised of three domains – the real (entities that have the power to activate mechanisms of action), the actual (events and their consequences that have been caused by mechanisms being activated) and the empirical (events or effects that have been observed and/or experienced) (52).

Our participatory approach embedded in a realist ontology avoids the making of false connections between the real and the empirical, which in practical terms means avoiding taking actions that are not based on lived-experience.

## Conclusions

This co-development approach has proposed some specific techniques to support the development of inclusive programme theories, ensuring these are embedded in the communities the intervention will be focused on. First, to enable full collaboration the presentation and development of theory leans on a range of approaches including illustrative vignettes. Second, PPIE and the involvement of stakeholders throughout the study provides multiple and ongoing opportunities for co-production, rather than just at occasional punctuations.

The paper therefore offers novel contributions to research teams developing initial programme theories by describing methods for connecting with and drawing on patient and public involvement and engagement. The approach can be applied to other studies wishing to draw on a range of knowledges, to initiate innovations and facilitation at the pre-intervention stage.

## Data Availability

The original contributions presented in the study are included in the article/**Supplementary materials**, further inquiries can be directed to the corresponding author/s.
