# Supplementary material for: Supporting care home residents in the last year of life through ‘Needs Rounds’: Development of a pre-implementation programme theory through a rapid collaborative online approach
Source: Front Health Serv. 2023 Jan 9;2:1019602. doi: 10.3389/frhs.2022.1019602 (PMC10012649; doi:10.3389/frhs.2022.1019602)
Supplement: Supplementary file 1 [file Datasheet1.pdf]

Supplementary File

Illustrative questions in interviews and workshops

**Example interview questions: Phase 1**	**Prompts in workshop breakout sessions**
Describe your care home (probes: number of beds, staff: resident ratio, funding source, independent/chain, number of staff, staffing skill mix e.g. nurses, is there a library or resource area for staff?, does your care home feel spacious? Estimated resident turnover rates).	What experiences or stories does the theory spark for you?
What is the process for new policies being introduced (who from? Discussion? How communicated?)	Does the stated theory make sense for your service?
How many formal meetings happen in the care home? (probes: management meetings, care planning meetings, service planning meetings, frequency etc)	Do the 'mechanisms' (the turning cogs which make change happen) map onto your understanding from what we've talked about in the other workshops?
What informal meetings happen? (e.g. is there a staff room, how often do staff interact with management informally)	Do the 'contextual' factors (the onion rings of wider policy, local service contexts) map onto what we've previously discussed? Is there anything important missing?
How would you describe staff confidence in looking after people approaching end of life, to keep them comfortable?	Do the outcomes (what we're hoping to achieve) map onto your understanding from the previous workshops?
What education/training and induction do staff receive on end of life care? And palliative care? And advance care plans?	Does the logic/chain of inference make sense? Can you see how the mechanisms described interact with the contexts to lead to the outcomes?
When we commence Needs Rounds, which staff do you expect to attend from here? (How many? What job role?)	Are there any outcomes, which don't have a clear mechanism?
How much do you think Needs Rounds will be prioritised?	Are there any mechanisms that don't take account of the context?
	Taking all 5 theories together, are there any gaps?
	Do we need to change anything in the context to help the NRs fit, and if so what? How can we work with local leaders to i) ensure they buy into Needs Rounds and perceive it to be

	valuable; ii) promote a collective shared view of Needs Rounds as valuable and worthwhile?
